# Editorial

**Published:** 1901-02-15

**Authors:** 


### EDITORIAL.

The *Dental Cosmos* for January sent out an edition of 39,000 copies, or one to every English speaking dentist whose name and address could be obtained. The *Dental Cosmos* has long held the distinction of the leading dental journal of the world, and we trust it may receive such support from the profession all over the world as it deserves, and that it may always keep pace with the progress of the profession, accomplishing that which no other journal has the facilities for doing.

The price of teeth has again advanced, because of the increased cost of platinum. Plain and gum teeth for vulcanite work are now fifteen cents each, and plate teeth are sixteen cents. Just what the effect of this continued increase in the cost of material is to be we can not tell. The diatoric or pinless teeth will doubtless come into favor for all kinds of plate work for the plastic bases. For solder work the platinum pins are essential and we shall have to use them. Perhaps it may stimulate some one to invent a low-fusing tooth porcelain that is sufficiently durable for practical use.

From observation of the methods employed by a majority of the dental practitioners one meets, and from reading the current literature, we are often forced to the conclusion that in many directions the practice of dentistry has in a very few particulars only become simplified to anything like a scientific practice, or even a generally accepted and practiced method. As an illustration, the editor of the *Items of Interest* has recently published a symposium of the opinions of a considerable number of teachers of prosthetic dentistry on the advisability of using the suction chamber in artificial dentures. And it is found that these teachers are nearly equally divided on the question of the use of this means for securing a more durable adaptation of upper plates. And what is equally surprising is that they don't even agree upon a name for the appliance. We do not doubt but that if a canvass were made of the profession we should find it equally divided. Are we not sufficiently intelligent to make a pronouncement on so simple a matter as this that shall be if not final, at least carry sufficient weight to establish a rule of practice for the guidance of the beginner and as a basis for judgment to such as may want to exercise intelligently a choice in such cases as experience would seem to demand a departure from the usual practice?

---

Is it not time for the practitioners of dentistry to systematize the practice of dental therapeutics? If we can not settle the "*Air Chamber*" question, or the "*Amalgam*" question, can we not, in view of all the light that has been shed on the subject of fermentation and putrefaction by the exhaustive researches made in the field of bacteriology, come to some definite conclusions as to the disinfection of dental caries, gangrenous pulps and alveolar abscesses? If so, we ought to intelligently apply adequate and proper remedies in a systematic way to overcome the destructive tendencies of infection. Our text-books are not intelligent on this subject, at least they present no systematic treatment of the subject; and judging from the product, teachers in our schools have no clearer conception of the subject. Neither do we find anything in our periodical literature.

or in the cases which fall into our hands from practitioners to give us hope that there is any uniform practice or anything that is worthy the name of a scientific system. In fact, it seems that there is very little effort made on the part of writers and practitioners to intelligently proceed, for instance, in the matter of disinfection. In reading the published papers with their discussions from our best societies on the subject of "*root filling*" one is compelled to admit that there is a woeful disregard of the laws of physics and chemical reaction, judging from the suggestions made. Is it not time we should remember and make use of some of the well established facts and base our procedure upon their laws? Water will seek its own level, it will not mix with oil, neither will it decompose into its elements at body temperatures. Acids and alkalies are pharmaceutically incompatible and are equally so physiologically, and usually therapeutically. Caustics and escharotics are very different substances therapeutically—although the same chemically; they are always destructive of the soft tissues and never restorative, and yet how often we hear them spoken of as curative remedies. Disinfectants and antiseptics are therapeutically very different substances, and yet they may chemically be identical. This confusion and variety of practice which we deplore must be inevitable, except we recognize distinctions and base our practice upon the drug and disease characteristics used in practice. Some things have been done and something is doing in the right direction, but it does seem as though we ought to make more rapid progress with our present light and the abundance of workers and facilities.

---

THE S. S. White Dental Manufacturing Company announces in the January *Cosmos* that it has withdrawn its membership from what is known as the Dental Trade Association, and gives as a reason that it could not retain its membership and maintain a branch house in one of the Southern States. It takes occasion to make the announcement that it is not in any sense a "trust concern," but is simply a corporation which has grown out of the business of the late Samuel S. White.

It also announces that it has no sympathy with the International Tooth Crown Company, and never has had any interest of any kind whatever in that concern.

It seems to us that, so far as the profession generally is concerned there has been no sufficient grounds for any of the censure which certain parties have attempted to cast upon the actions of this corporation. Every fair-minded man in the dental profession who thinks enough of his profession to strive by his individual efforts to raise it above the level of the tinkerer and charlatan, knows how much he owes to the men who have furnished him with the facilities which have enabled him to devote his energies to the more professional branches of his calling; and if he has any gratitude he will most heartily commend and be willing to reward every effort made to supply him with superior facilities. We personally are most happy to express our gratitude to this great organization, be it a trust or what not, for the acceptable way in which it has ever met our personal wants. And we doubt not that this sentiment will meet with a hearty endorsement from a large majority of the best in our profession. If we have paid high prices for goods manufactured by this house, we have always enjoyed the comfortable feeling that we were utilizing the best helps attainable and that our patients were so much the better advantaged. We sincerely trust that the policy of this house in the past, to make only the best that could be produced, may be continued, for we are confident that its past success was attained only upon this fact being recognized generally by the profession.

